# Closing the Loop with Keratin-Rich Fibrous Materials

**DOI:** 10.3390/polym13111896

**Published:** 2021-06-07

**Authors:** Simona Perța-Crișan, Claudiu Ștefan Ursachi, Simona Gavrilaș, Florin Oancea, Florentina-Daniela Munteanu

**Affiliations:** 1 Faculty of Food Engineering, Tourism and Environmental Protection, “Aurel Vlaicu” University of Arad, 2-4 E. Drăgoi Str., 310330 Arad, Romania; simona.perta-crisan@uav.ro (S.P.-C.); claudiu.ursachi@uav.ro (C.Ș.U.); simona.gavrilas@uav.ro (S.G.); 2 Bioresource Department, National Institute for Research & Development in Chemistry and Petrochemistry-ICECHIM Bucharest, 202 Splaiul Independentei, 6th District, 060021 Bucharest, Romania; florin.oancea@icechim.ro

**Keywords:** keratin-rich side-streams, valorization, keratin extraction methods, applications

## Abstract

One of the agro-industry’s side streams that is widely met is the-keratin rich fibrous material that is becoming a waste product without valorization. Its management as a waste is costly, as the incineration of this type of waste constitutes high environmental concern. Considering these facts, the keratin-rich waste can be considered as a treasure for the producers interested in the valorization of such slowly-biodegradable by-products. As keratin is a protein that needs harsh conditions for its degradation, and that in most of the cases its constitutive amino acids are destroyed, we review new extraction methods that are eco-friendly and cost-effective. The chemical and enzymatic extractions of keratin are compared and the optimization of the extraction conditions at the lab scale is considered. In this study, there are also considered the potential applications of the extracted keratin as well as the reuse of the by-products obtained during the extraction processes.

## 1. Introduction

The etymology of the “keratin” term comes from the Greek word “kera”, which means “horn”, referring to the description of the material which forms hard tissues in animals and that was formulated for the first time around 1850 [1].

Keratin is a structural protein encountered in epithelial cells of most vertebrates [2], representing their most abundant and important biopolymer, after collagen [3]. Keratin is a rigid, fibrous protein produced in the epidermal layer or outer covering of vertebrates’ body [3,4], being the third most abundant polymer found in nature after chitin and cellulose [1].

Keratin is frequently wrongly understood as a single substance, even if it represents a complex mixture of proteins, such as keratins and keratin-associated proteins [4,5]. Keratins are filament-forming, high-sulfur content proteins and belong to the group of intermediate filament proteins (IFs) [6,7], which are insoluble in ordinary protein solvents and also undigested with pepsin or trypsin [1].

Keratin proteins are formed through a systematic biological process of cellular differentiation which converts living cells of the epidermis into cornified, structurally stable, and without metabolic activity cells [4,8]. The resulted dead cells build up at the outermost layer of skin during the keratinization process [2]. Keratinous materials are composed of intracellularly synthesized keratins [4], meaning cells filled with keratin because of keratinization which substitutes the cytoplasmatic content of cells with filamentous proteins [7]. Keratin-producing cells die after producing process, thus leading to the appearance of non-vascularized tissue [3].

Keratin biomass is provided by living organisms or dead animals’ body. It includes hair, skin, nails, feathers, wool, horns, hooves, and scales, as major sources of keratins, that can be extracted from wool and hair, horns, and hooves [9]. Keratin biomass possesses a high amount of protein [10]. Thus, the chicken feathers dry matter contains 90% crude keratin [11], the wool up to 95% by weight, while keratin content in human hair is about 80% [9].

Some industries, such as the food industry, wool industry, and slaughterhouses produce keratin biomass in large amounts, meaning millions of tons [9]. The most significant quantities of keratin can be found in keratinous wastes generated by poultry, bovine, and pork meat productions, in the form of feathers, animal bristles, and horns [10,12]. Some amounts of keratin also result from wool and leather industries and hair salons. The total estimated quantity of keratin-containing wastes reaches 40 million tons annually [13,14]. Transforming the ovine skins and bovine hides into leather caused about 2 × 10^5^ tons of hair and 5.6 × 10^4^ tons of wool per year [15,16], while domains such as textile and apparels generate 2.5 million tons of keratinous wastes yearly, in the form of waste and used wool materials [12]. Among keratinous materials, the most abundant and sustainable in nature is the chicken feather waste [17]. Globally quantity of feathers produced by poultry processing, as a by-product, was estimated at ~8.5 billion tons per year [1,18], while annual worldwide production of poultry encounters a continuous increase, anticipating reaching more than 24.8 billion animals in 2030 and 37.0 billion in 2050, respectively [10,19]. 

Closing the loop with keratin-rich side streams prevents the generation of the keratin wastes. Keratin-based wastes elimination is a serious problem, because of the damages caused to the environment by their incineration or disposing-off in landfills. Slow degradation of the keratin waste dumps promotes saprophytic development of the dermatophytes and is considered a potential health hazard [20,21]. In the case of burning, the sulfur content is culpable for pollution increasing, while discharging in the ecosystem can lead both to landscape degradation and soil and groundwater contamination [13,22,23,24]. A special interest is accorded to the management of unmarketable wool, which is an increasing waste in many countries. The low capacity of biodegradation and the long-term environmental pollution are the major worries in this regard [15]. For minimizing environmental pollution, there is a real interest for keratinous side streams to be converted into high-value consumable products [1,25], with both environmental and financial benefits.

The present review focuses on keratin and the possibilities of its valorization from different wastes. The study details keratin’s composition and properties, as well as the various “green” methods used for its extraction. Moreover, the applications of the extracted keratin in different fields are also considered, including perspectives of the disposal and/or reuse of the by-products obtained during the extraction processes. 

## 2. Composition and Structure of Keratin

The structural units of keratin are 20 amino acids, united by varied inter and intramolecular links consisting of hydrogen, disulfide, hydrophobic, and ionic bonds, thus leading to increased mechanical strength and stability of keratin structure and keratinous materials [1,26,27,28]. The high stability, resistance, and insolubility of keratin are due to the network structure created by the numerous strong, covalent disulfide bonds between thiol (-SH) groups contained in cysteine residues, within and between polypeptides of keratin [10,23,29,30,31]. The formed structure induces compactness to keratin, because of the network created by the adjacent polypeptides and sulfur–sulfur cross-links [17,32,33]. On the other hand, these disulfide linkages avoid releasing some helpful amino acids and short peptides found in keratin [34,35].

Keratin is a fibrous protein with high content of cysteine in the amino acid sequence. Relative to other structural proteins, keratin presents a higher content of cysteine in its structure, ranging between 7–13% [36]. Besides cysteine, which is responsible for disulfide bonds that cross-link protein chains, keratin also includes important amounts of other amino acids such as arginine, glycine, serine, proline, glutamic acid, and aspartic acid and the essential amino acids valine, leucine, and threonine. Low quantities are registered for histidine, lysine, and methionine. Glycine confers hydrophobicity, rigidity, and degradation resistance, and together with cysteine is responsible for keratins strength, low water solubility, and resistance to biological degradation, due to their presence in high levels. Glycine is present in high amounts especially in rigid keratinous materials, such as claws or beaks, where its level exceeds 28% [10]. Keratin’s solubility in water may be improved by using the heat accompanied by a reducing agent when a mild and acidic pH is present [17,37].

The structure of keratin is based on polypeptide chains, which can curl into helices in the α-conformation or configure into pleated sheet arrangements in the β-conformation [3]. Depending on their structure, keratins are differentiated in the literature as α-keratins and β-keratins [36]. Keratins have also been described the γ-keratins [38]. These distinct configurations are possible due to the differences registered within keratins’ molecular structure and in their filaments’ formation [7]. In regard to the α- and γ-keratins from hair, their special properties can be revealed only by using further advanced and laborious separation and purification methods of the crude extracts of keratin [38]. 

### 2.1. α-Keratin

The structure of α-keratin consists of two right-handed helically wrapped polypeptides chains, stabilized by hydrogen bonds [7]. The two sub-filaments, that coil together, are different, namely the Type I (acidic) with molecular weights of 40–50 kDa and the Type II (basic or neutral) with 55–65 kDa molecular weights [1,10,39]. The assembling of two α-helix chains leads to the formation of disulfide links and thus, to a left-handed coiled-coil heterodimer. Next, these dimers assemble end-to-end and side-by-side by disulfide bonds and form a protofilament with a diameter of ~2 nm. By lateral association of two protofilaments is formed a protofibril and by grouping four protofibrils result in the intermediate filaments (IFs) with a diameter of 8–10 nm, which form the bulk of cytoskeleton and epidermal appendages (hair, horns, feathers, wool, and nails) [1,36]. IFs are embedded in the keratin matrix, an amorphous and rich in sulfur mass, characterized by cysteine-rich protein chains and high quantities of tyrosine, glycine, and phenylalanine residues [7,40]. α-keratins presents durability, resistance, and insolubility, at the same time constituting a danger for the environment due to α-helix chains which are resistant to microbial decomposition [1,18]. The constitutive amino acids of α-keratins are hydrophobic, namely methionine, valine, alanine, isoleucine, and phenylalanine [1]. When stretched, the α-helices change into β-pleated sheets [31], this form being reversible up to about 30% strain [3].

### 2.2. β-Keratin

β-keratin is a functional protein whose filament molecular unit is a pleated sheet [7]. In the first phase, the formation of β-keratin filament implies four lateral β-strands, constituted because of polypeptide chain folding in its central region, which then links through intermolecular hydrogen bonds [7,41] and produces small rigid planar surfaces lightly bent together in the form of a pleated arrangement [3]. This structure is stabilized, on the one hand, by the hydrogen bonds between β-strands which form a sheet and by the planarity of peptide bonds which force the pleating of the β-sheet, on the other hand [7]. In the second phase, two pleated sheets are distorted in a left-handed helical surface superpose and roll in contrary directions, leading to the constitution of a filament with a 4 nm diameter. The peptide chains’ terminal parts wrap the filaments, forming the matrix [7,41]. β-keratin presents a high concentration of cysteine, that facilitate the creation of disulfide bonds which are responsible for its oxidation resistance and stiffness. The molecular weight ranges between 10–14 kDa [1].

Bin Wang et al. [7] summarized the specific features of α-keratin and β-keratin, pointing out the differences registered in their structure (Table 1), in addition to the structural characteristic which is a filament-matrix one in both cases: IFs (α-keratin) and β-keratin filaments (β-keratin) incorporated in an amorphous matrix.

### 2.3. γ-Keratin

This type of keratin was described one decade ago, and it refers to the globular proteins initially described as keratin-associated proteins in the (human) hair. Such proteins are rich in sulfur amino acids and possess lower molecular mass (from 10 to 15 kDa) compared to the other types of keratins [38]. Their function is to cross-link the keratin intermediate filaments (KIFs) and are involved in the interaction of KIFs with the cytoskeleton and cellular membranes [42]. The γ-keratin class was demonstrated to include two subclasses of globular proteins, one sub-class with a small molecular mass (7 kDa–9 kDa), rich in glycine and tyrosine, and the other one rich in sulfur, with a higher molecular mass, ranging from 10 kDa to 35 kDa [43]. The wool contains less γ-keratin than human hair [42]. γ-keratin is less susceptible to protease hydrolysis [44]. γ-keratin promotes cell rescue after thermal injury in vitro and is more suitable for the preparation of wound dressing products [45].

### 2.4. Classification of Keratins

Classification of keratin is accomplished considering some distinct factors: X-ray diffraction and sulfur content or physical and chemical properties [1]. Moreover, there is one more possibility of classifying keratin from terrestrial animals, depending on its provenience: mammalian keratin, reptilian keratin, and avian keratin [7]. In fish, keratin is present not only in the epidermal cell, but also in mesenchymal tissues [46]. A potential interest can be accorded to the keratin from fish mucus, which could bound and agglutinate fungal cells [47].

Depending on the characteristic X-ray diffraction pattern, two types of keratin are distinguished: α-keratin (α-pattern) and β-keratin (β-pattern) [31]. α-keratin mainly occurs in the vertebrates’ epithelium (stratum corneum) and some other mammalian epidermal materials, such as wool, fingernails, horns, hooves, quills, and hair [1,7,10]. Approximately 30 variants of α-keratin are found in these materials [3]. All keratins encountered in mammals are α-type, while birds and reptiles can produce both α-type and β-type keratins [31]. α-keratin induces mechanical strength to epithelial cells in feathers and reptilian scales, adhesiveness, and stretching malleability, due to its presence in the epidermis placed between scales [10,48,49]. On the other side, β-keratin found in reptilian scales leads to protection due to its major hydrophobicity and microbiological resistance, even though it presents restricted extensibility [10,50]. β-keratin, which is tougher than the α-type, is present exclusively in avian and reptilian tissues, such as feathers, avian beaks and claws, and reptilian claws and scales [3,7,10,36]. Some keratinous materials are based both on α- and β-keratin, namely the reptilian epidermis, the hard and soft epidermis of Testudines’, and pangolin scales [7].

According to the biosynthesis mechanism and sulfur content of keratins, those can be classified into soft and hard keratins [7,31,51,52]. “Soft” and “hard” are words that describe only tactile sensations, but the differences registered in the chemical composition of these keratins are considerable [4,53,54]. Usually, soft keratins contain a lower quantity of sulfur than the hard ones, are not so consolidated and more flexible. They can be found especially in the skin (stratum corneum) and possess less than 3% of sulfur [1,3,7,55,56]. Skin chemical composition is characterized by a sulfur amount of about 1% (dry weight), uniformly distributed between cysteine and methionine, amino acids which form proteins by combination. The lipids from the skin consist of fatty acids, phospholipids, and sterol, and represent about 4% of dry weight [4]. Hard keratins, with a sulfur content of more than 3%, confer a more coherent and tough structure to keratinous materials which incorporate them, such as wool, feathers, horns, nails, hair, beaks, claws, and quills [1,3,7,55,56]. The sulfur content of hard keratins is provided by high sulfur proteins formed mainly through cysteine combination. Other non-protein components, such as lipids or glycogen, are quantitatively negligible in hard keratins [4]. 

### 2.5. Properties and Functions 

Keratin is one of the toughest biological materials, even if it is formed exclusively of natural polymers and rarely contains minerals [7,57]. The mineralization with calcium or other salts contributes to its hardening [7]. 

Keratinous materials possess some specific properties such as durability, toughness, and lack of reactivity to the environment, these conferring protection and mechanical support to vertebrates in their adaptation to the external environment [7,55]. The mechanical durability of keratin is due to its structure, which is abundant in disulfide cross-links, hydrophobic bonds, and hydrogen connections [26,58,59,60]. Because keratins are the forming units of cellular intermediate filaments (IFs), they can supply mechanical support to epithelial cells and provide stability between epithelial cells and epithelial cells fixation to the basal membrane [61]. Mechanical properties of keratinous materials are influenced by the orientation, packing and volume fractions of filaments, while mechanical comportment is affected by hydration, considering their strain sensitiveness on humidity. Thereby, the increase of humidity produces the decreasing of strength and Young’s modulus values [62]. 

Keratin proteins are produced in the integument, which represents organisms’ protective coating and is formed by two distinct parts, namely the dermis and epidermis. The main constituents of the dermis are elastin and collagen, while the epidermis, which constitutes the outer layer, is made of epidermal cells [2]. For mammals and fowl, keratin constitutes the bulk of the corneous stratum of the epidermis and epidermal appendages such as horns, nails, hair, claws, beaks, and feathers [7], and at the same time a major part of their protective matrix [4,53]. Thus, the main role of keratin is to make these epidermal materials an insoluble and unreactive barrier against the environment [4]. 

The excellent mechanical functions of keratinous fibrous materials, both in tension (e.g., wool) and compression (e.g., hooves) [3], depending on the provider animal, respectively, on the complex hierarchical structures which include filament-matrix structures at the nanoscale and varied arrangements of keratinized cells at micro and macroscales. This induces different mechanical properties, with values for Young’s modulus ranging from 2 MPa in stratum corneum to 2.5 GPa in feathers and tensile strength from 2 MPa in stratum corneum to 530 MPa in dry hagfish slime threads. Thus, keratin-based materials can accomplish diverse functions, such as diffusion barrier, penetration resistance, external attack buffering, buckling resistance, and energy absorption [7]. Because of different possible morphologies, keratinous materials possess a series of distinct functions in organisms: compact waterproof stratum (osteoderms and skin), compact shells filled with a poriferous material that confer light and rigid structures (feathers, beaks, and quills), hard blocks with embedded tubules which confer impact resistance (horns and hooves), and filamentary structures (gecko feet and hagfish slime) [3].

In addition to mechanical resistance, keratin and keratinous materials possess intrinsic biocompatibility and biodegradability, with major importance for the domain of modern biomaterials [1,36].

## 3. Methods for the Valorization of Keratins by Extraction

Keratin is extremely insoluble in water and organic solvents and thermally and chemically stable due to the inter- and intramolecular hydrogen bonds and strong disulfide bridges [63]. Therefore, the strategies for extracting and dissolving the keratin from various natural tissues consist of cleaving the disulfide and hydrogen bonds, in order to determine its conversion into a non-cross-linked form and thus enhancing its water solubility. Several methodologies of keratin extraction developed over the years are available in the literature. These can be classified by several criteria as it follows:

- Considering the effect on the native structure of keratin extraction methods can be grouped in protected solubilization (extracted keratin molecules are approximately intact) and unprotected solubilization (molecular structure of keratin is destroyed by degrading peptide bonds, disulfide bridges, and intermolecular hydrogen bonds resulting in a low molecular weight proteins and polypeptides mixture) [64,65];
- About the used extraction methods there are chemical (reduction, oxidation, hydrolysis, sulfitolysis, and the use of ionic liquid), physical (steam explosion, superheated water treatment, and microwave irradiation), and biological (microbial and enzymatic) methods [66];
- Regarding the effect on the environment, some green methods have been developed, such as microwave irradiation, supercritical water extraction, and steam explosion.

Considering the distinct solubility of α-keratin and β-keratin, Wang et al. [67] summarized the methods proved to be suitable for their extraction from specific keratinous materials, in order to obtain keratin fractions in satisfactory amounts. Thus, in the case of α-keratinous materials were efficiently applied reduction, oxidation, and sulfitolysis procedures, while for β-keratinous ones proved to be adequate the alkaline thioglycolate and the combination of a disulfide bond cleaving reagent with a protein denaturant.

We will further highlight the characteristics of the main extraction methods currently used or applied within research studies.

### 3.1. Chemical Methods for Keratin Extraction

Keratin extraction by chemical hydrolysis (acid, base, and catalyst) requires high temperature and pressure. The acidic extraction of keratin can be conducted through the use of different acids, such as hydrochloric acid, sulfuric acid, and peracetic acid.

The extraction of keratin performed in the presence of hydrochloric acid implies high concentration (usually 12 M) of hydrochloric acid, high temperature (110 °C), and a long hydrolysis time (12 h) [68]. 

#### 3.1.1. Oxidative Extraction

**Figure 1:**
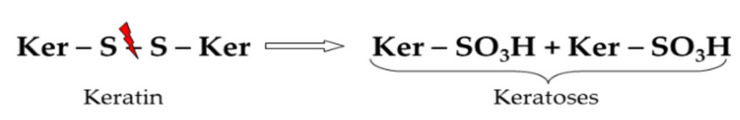
Oxidation of keratin into cysteic acid residues (keratoses).

As reported by Earland et al. [69], since 1955, the oxidative methods are one of the oldest techniques used for keratin solubilization. Most studies of oxidative methods are focused on the extraction of keratin from wool and hair. Oxidation agents such as peracetic acid, hydrogen peroxide, performic acid, or potassium permanganate can partially break the disulfide bonds bridges from the keratin tissues and oxidize cystine to cysteic acid residues (Figure 1) [65]. The oxidized keratins are referred to as keratoses, are chemically modified and may be extracted and separated sequentially from the keratin source into different fractions (α-, β-, and γ-keratose), based on their solubility at different pH values [70,71,72].

The disadvantages of the oxidative methods consist of long extraction times, require large quantities of oxidizing agents, and often has been reported that only a part of keratin is solubilized. Furthermore, keratoses are also susceptible to degrade relatively quickly by hydrolytic degradation [38,73]. Some of the optimized conditions for oxidative methods, available in the literature, are presented in Table 2.

#### 3.1.2. Reductive Extraction 

**Figure 2:**
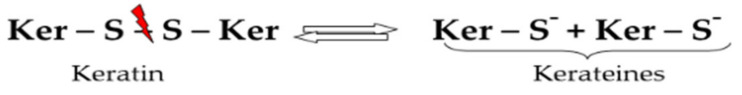
Reduction of keratin into kerateines.

The disulfide linkage in the polypeptide chain of keratin macromolecule can be reduced by thiols such as thioglycolic acid, 2-mercaptoethanol, and dithiothreitol. Thiol anions of reducing agents, formed in alkaline media, generate double nucleophilic substitution and produce soluble keratin forms known as kerateines (Figure 2). To improve kerateines water solubility, by breaking the hydrogen bonds between polypeptides chains most of the reductive methods are carried out using urea or thiourea as denaturing agent [77,78]. Furthermore, extraction rate and kerateines stability can be increased by using dodecyl sulfate (SDS) as a surfactant. Yamauchi et al. reported that the use of urea and SDS, accelerated the keratin extraction from wool, increased the process yield, and prevent the kerateines chain aggregation by blocking the formation of new linkages [44]. In another study, Schrooyen et al. [79] found that the addition of SDS to the keratin solution prevents the oxidation between different keratin chains and as a consequence intermolecular disulfide bonds formation and protein agglomeration. Reducing agents such as mercaptoethanol presents some important disadvantages due to their toxicity and harmful effects on the environment [23]. A less toxic alternative to the extraction with mercaptoethanol is to use the acidic extraction with Tris(2-carboxyethyl)phosphine (TCEP) in the presence of urea, thiourea, and Tris. The acidic conditions are assured by the hydrochloride form of TCEP [80]. Another advantage of this method is due to the water solubility of TCEP, its increased stability in solution, and the absence of odor.

Some of the reductive agents and the processing conditions are presented in Table 3.

#### 3.1.3. Sulfitolysis

**Figure 3:**
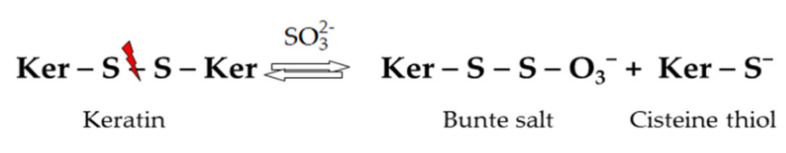
Sulfitolysis of keratin.

The disulfide bonds are cleaved by sulfites and bisulfites resulting in cysteine thiol and cysteine-S-sulphonate anion or Bunte salt (Figure 3) [83]. Ramya et al., in their comparative study of keratin extraction from red sheep’s hair by different methods (oxidative, alkaline, and reductive) considered that sulfitolysis was most effective, nonhazardous, and produced the maximum yield [82]. According to several authors, sulfitolysis is a better alternative than the standard reductive methods for keratin extraction since sodium sulfites and bisulfites are not so toxic to the environment and more economic. Depending on the keratin source the extraction yield can be improved by using protein denaturing agents (urea), surfactants (SDS), and by adjustment of processing parameters (Table 4). 

#### 3.1.4. Alkaline Extraction

Heating in concentrated alkali solution causes pronounced irreversible hydrolysis of keratin macromolecule by breaking of the peptide bonds, primary amide bonds, and cystine disulfide bonds [85]. The breakdown of these bonds leads to a soluble oligopeptide fraction and a solid residue but also the formation of the unpleasant alkaline sulfide odor. Alkaline methods require high amounts of alkali reagents and also, high amounts of acids to neutralize and precipitate the solubilized keratin fraction [86]. The yield and the stability of the keratin hydrolysate depend on the conditions used for hydrolysis (temperature, reaction time, type, and concentration of alkali and acid used) [66,87], as shown in Table 5.

#### 3.1.5. Extraction with Ionic Liquids and Deep Eutectic Solvents 

Ionic liquids (ILs) are a group of salts that usually have a low melting point (below 100 °C), composed of an organic cation and some organic or inorganic anions [89]. Due to their unique properties such as chemical and thermal stability, miscibility with other solvents, high solvation, environmentally friendly, low vapor pressure, low volatility, and non-flammability, these are used for a wide variety of applications [90,91,92]. The most studies indicate that ILs of imidazole such as 1-allyl-3-methylimidazolium chloride [AMIM]Cl, 1-Ethyl-3-methylimidazolium chloride [EMIM]Cl, 1-butyl-3-methylimidazolium chloride [BMIM]Cl, 1-allyl-3-methylimidazolium dicyanamide [AMIM][dca], 1-Ethyl-1,5-diazabicyclo[4.3.0]-non-5-enium diethyl phosphate [DBNE]DEP, and 1-methyl-1,5-diazabicyclo[4.3.0]non-5-enium dimethyl phosphate ([DBNM]DMP) are frequently used for protein dissolution since they have demonstrated the best extraction capacity [1,89,93]. Dissolution of keratin from different biomasses by ILs has been explored by several authors, due to their potential to better conserve the protein integrity and the possibility of being regenerated and reused several times [89]. Idris et al. [94,95] investigated different ILS, including [BMIM]Cl, [AMIM]Cl, [AMIM][dca], and choline thioglycolate for the dissolution of wool keratin. The results demonstrated that [AMIM][dca] had a higher extraction level of keratin (475 mg/g) and the addition of mercaptoethanol as a reducing agent increased the wool solubility up to 575 mg/g. It was also reported that the dissolution took place without a significant change of the protein backbone but depending on processing parameters was possible the scission of the polypeptide chains into smaller segments [71,95].

The temperature has an important role in keratin dissolution by ILs regarding the extraction yield and the structure of proteins. Gosh et al. [71] evaluated the relationship between temperature and these two parameters by extracting the keratin from wool with [BMIM]C. The study showed that temperature increasing from 120 °C to 180 °C reduced the keratin yield from 57% to 18% and the researchers concluded that this can be attributed to the formation of water-soluble low molecular mass peptides and free amino acids [71].

The most used ILs for keratin extraction and the processing conditions are presented in Table 6.

Other promising solvents for extraction and separation of biological compounds are the deep eutectic solvents (DES). They are considered an alternative to ILs for the dissolution and conversion of keratin biomass due to their favorable properties such as lack of toxicity, cost, and the possibility to adjust their properties [99]. DES are low transition temperature mixtures consisting of two components: hydrogen bonding donor (HBD) and hydrogen bonding acceptor (HBA). HBA and HBD interact with each other to form a stable solvent at a low temperature. The HBD are usually amines, carboxylic acids, polyols, carbohydrates, or acid amides while the HBA is a quaternary ammonium salt [99,100,101]. In the last years, there were reported few studies of different DES employed in the extraction of proteins. Wang et al. [67] used the combination of choline chloride and oxalic acid with a molar ratio of 1:2 as DES for dissolving keratin from rabbit hair. The authors reported a total dissolution of rabbit hair at 120 °C after 2 h of heating. The molecular weight obtained for keratin was ranging from 3.8 to 5.8 kDa, comparable to the conventional methods [67]. In another study, Sakhno et al. [102] measured the solubility of rabbit hair in a mixture of two DES in a different composition. DES 1 consisted of a combination of choline chloride and urea and DES 2 in a combination of choline chloride and urea-hydrogen peroxide adduct both with the molar ratio of 1:2. The extraction was carried out for 10 h at a temperature of 30 °C. Results showed that the mixture of DES 1 and DES 2 (1:2) had the highest solubility (79%) for the rabbit hair [102].

### 3.2. Enzymatic and Microbial Methods

Keratins are resistant to biodegradation by common proteolytic enzymes, such as trypsin and pepsin, due to their high degree of cross-linking sustained by disulfide bonds and hydrophobic interactions of the polypeptides [36]. Nevertheless, large strains of microorganisms including bacteria, actinomycetes, or fungi can produce keratinases, a group of proteolytic enzymes that can hydrolyze keratin to produce soluble proteins [87,103].

Keratinases (EC 3.4.21/24/99.11) belong in general to the serine or metalloproteinases’ group and are classified as neutral or alkaline enzymes with optimum activity at values of the pH ranging between 6.0 and 9.0 [104,105]. Regarding temperature, the optimum interval for their function is between 45 and 60 °C [106]. The mechanism of keratin degradation by microbial keratinases is not fully known. One of the hypotheses accepted by many researchers considers the catalytic activity of keratinases a two-stage process of keratin degradation: sulfitolysis and proteolysis. In the first stage, disulfide bonds between polypeptide chains are cleaved by reductase, thereby thiol groups are liberated, and keratin conformation is changed. In the second stage, denatured keratin is decomposed by protease resulting soluble peptides and amino acids [104,107,108]. Biological hydrolysis of keratin is very attractive because involves mild operation conditions, less energy, and constitutes a green, environmentally safe method [1]. Under laboratory conditions, several microbial species showed their ability to metabolize wool, feathers, or other keratin sources. Some of the keratinolytic microorganisms reported in the literature as well as their optimal condition used to degrade keratin by-products are presented in Table 7.

Lytic polysaccharide monooxygenases (LPMO), which are breaking down recalcitrance of cellulose and chitin were hypothesized to be involved also in keratin degradation because are present in the genome of fungi degrading keratin-rich biomaterials, such as *Onygena corvina* [122]. LPMO were proven to be auxiliary enzymes, which probably promote the action of the keratinases by destabilization of the keratin structure [104]. The contribution of LPMO to keratin structure destabilization could be related also to their contribution to disulfide bridge oxidation due to in situ production of reactive oxygen species [123].

### 3.3. Physical Methods for Keratin Extraction

#### 3.3.1. Steam Explosion Extraction

Steam explosion is a hydrothermal treatment of biomasses that uses high temperature saturated steam (180–240 °C), under pressure (1–3.5 MPa), and for a short timeframe [1]. The process continues with rapid restoration of pressure to atmospheric value obtaining an explosive decompression that causes an advanced structural rupture of the biomass [37]. Keratin sources subjected to steam explosion led to a mixture of low molecular mass soluble peptides and free amino acids. Although the steam explosion method has a low environmental impact, reduced processing time, and low cost the high temperature and pressure during the process destroyed cysteine and reduced the quality of the final product [72]. Some of the optimal conditions for steam explosion methods are described in Table 8.

#### 3.3.2. Microwave Treatment

Microwave technology has been developed in the last years as an alternative heating method. The advantage of microwave is a reduced reacting time and an important energetic economy since heating is faster due to the internal heat generation and homogeneous rise of temperature for all the polar molecules present in the reactor [36]. Microwave-assisted heating has been applied by several authors for keratin extraction from different biomasses. Zoccola et al. [64] used microwave treatment to solubilize keratin from wool. In their work microwave irradiation with power ranging between 150–570 W was applied for 60 min to wool samples, at different temperatures (150; 170; 180 °C), and a maximum extraction yield of 62% was obtained at 180 °C. Extracted protein fraction was characterized by a very low molecular mass, among 3–8 kDa but the major drawback associated with this technique was that higher temperature-induced significant cystine loss which increased up to 99% at 180 °C. Lee et al. [127] combined microwave treatment and alkali hydrolysis for the extraction of keratin from feathers and considered it more efficient compared to the conventional heating-alkali method. It was reported that the maximum extraction yield of around 26 mg/mL protein was obtained by using a power of 800 W for 10 min, and 0.5 M sodium hydroxide by a liquor ratio of 1:50 [127].

#### 3.3.3. Superheated Water

At high temperature and pressure, water leads to the degradation of the keratin into oligopeptides. In their study, Bhvasar et al. [88] applied superheated water treatment (fiber, liquor ratio 1:3; 170 °C; 7 bar; 1 h) for extracting keratin from wool. The authors reported that almost all wool fibers were dissolved, and the resulted hydrolysates were composed of low molecular weight polypeptides and proteins with a low amount of cystine and a mainly disordered secondary structure. The results were comparable with the standard alkaline hydrolysis, but superheated water extraction is considered more economical and ecological [88]. In another study, Rajabinejad et al. [81] showed that the yield of extracted keratin from wool by superheated water treatment (fiber-liquid ratio 1:35; 170 °C; 30 min) was quite similar to oxidative, reductive, or sulfitolysis techniques. Although it is cheap, ecological, and easy to implement, the main disadvantage of this method is a high decrease of resulted peptides’ molecular mass and the loss of the temperature-sensitive amino acids [81]. In a recent study, Tasaki et al. [128] reported a novel technique for extraction of keratin from hog hairs, by combining thermal hydrolysis with ultrafiltration, for recovering keratin hydrolysate, and removing impurities from the reaction solution. The method consists of two-steps heat treatment of keratin source. The first step presumes heating at 140 °C to denature keratin in the intermediate filaments by breaking hydrogen bonds while the second step, which takes place at a higher temperature (140–220 °C) is to cleavage the disulfide bonds and solubilize the keratin protein. The experiment demonstrated that the recovery yield for the two-step heating process was higher than for the one-step process (without heating pre-treatment) and comparable with the chemical conventional methods [128]. The reduced processing time and operation cost, the absence of chemicals, and the environmental low impact constitute the main advantages of the superheated extraction method and make it available to the industrial-scale implementation.

## 4. Applications of Extracted Keratin

The keratinous waste produced by different industries (food, textile, and leather industry) requires a good management strategy for recycling and reuse.

Keratin has physical, chemical, and biological properties that make it the most important biopolymer of animal origin, right after collagen [3].

Knowing its characteristics, keratins represents an interesting material, prone to be used for cosmetics [128,129,130], biomedical [36,42,73,131,132,133,134,135,136], environmental [34,90,103,111,112,113,117], and agricultural applications [137]. As a base for biomaterials, the most exploited properties are referring to the ability to self-assembly, possession of cell-binding residues (glutamic acid, aspartic, acid, serine, leucine, aspartic acid, and valine) [133], since that is leading to a three-dimensional structure favoring the cellular attachment [36].

Considering the extensive number of applications of keratin and the development of its extraction methods, nowadays the forms in which the keratin is extracted are directly linked to the desired use. As a result, the keratin-based biomaterials are fibers, films, hydrogels, scaffolds, and sponges [1,42]. The formulation of cosmetics for the improvement of skin physiology is based on bioactive peptides. Super-adsorbent composites produced from extracted-keratin are used for the formulation of the agricultural inputs, such a sprayable mulch [138] or controlled released fertilizers [139].

Various methods for the extraction of keratin from keratin-rich waste are reported in the literature [17,26,62,65,67,72,74,76,78,80,82,84,96,128,136,140,141], but the viability of the keratin extraction method at an industrial scale and its impact on the environment should constantly be evaluated.

In general, the oxidative methods lead to the obtaining of keratoses, and different fractions of α-, β-, and γ-keratin can be separated according to their solubility. These are usually valorized for biomedical applications [36,42,72,131,132,142,143], such as wound healing [135,143,144,145,146] and tissue engineering [14,46,82].

Through the keratin extraction methods that are based on sulfitolysis there are obtained sulfo-kerateines with biomedical applications [38,78,147]. The low molecular weight mass proteins/peptides for cosmetic application are usually obtained through enzymatic or microbial hydrolysis [109,129,148].

The valorization of keratin-rich materials for further use in environmental applications, such as removal of heavy metals, is not only relying on the extraction of keratin from these materials but also on the chemical modification of the used materials, to increase the number of the functional groups that are necessary for the designed purpose [149]. In other cases, keratin was extracted by using a reducing agent [13,63,150] and thereafter cross-linked with glutaraldehyde for obtaining new materials that possess the capacity to remove recalcitrant pollutants from the environment [43].

For agricultural inputs of the keratinous wastes, a detailed overview is presented in Table 9. For emerging applications, the used keratin is generally obtained through hydrolysis [151] or sulfitolysis [12].

### 4.1. Cosmetic Applications

The most known applications of keratins are for cosmetics, especially for hair treatments [152], but beneficial effects were also observed for the improvement of the hydration and elasticity of the skin if a solution of hydrolyzed keratin peptides (<1000 Da) is used. Moreover, the hydration is held due to the reinforcement of the skin barrier’s integrity [129].

Recently, special attention was paid to the bioactive keratins [128,130], especially those with low molecular weight (LMW) [148]. Yeo et al. [148] reported the obtaining of low molecular weight (LMW) keratins through the anaerobic digestion of the native chicken feathers in presence of *Fervidobacterium islandicum AW-1*. They observed that the LMW keratins (<1 kDa) proved radical scavenging capacity and inhibit collagenase. Moreover, these keratins also showed to be good candidates for protection against the skin ageing induced by the ultraviolet radiation, UV-B [148].

Tinoco et al. [153] prepared a cosmetic formulation for hair in which keratin was mixed with a protein derived from maize, zein [153] that can steadily release perfumes on hair. Once applied to hair, the product forms a film-like structure over the hair fibers, and the perfume is released depending on the temperature and the physicochemical properties of the fragrances that are used. Other beneficial effects of this product refer to the improvement of hair hydration and its mechanical resistance.

The keratin proteins showed good bioactivity and biocompatibility which promotes them as excellent biomaterial to be used for biomedical applications.

Kim et al. [145] improved the solubility of the extracted human hair keratin by cross-linking with poly(ethylene glycol) and tyramine. Moreover, this modification also contributed to the in situ hydro-gelation [145]. The obtained hydrogel proved to enhance the in vivo wound healing in the case of a mouse with a full-thickness skin defect.

### 4.2. Biomedical Applications

In another study, extracted keratin and hyaluronic acid were incorporated in polyethylene oxide polymers and poly(ε-caprolactone) and afterwards were produced fibers through the emulsion and coaxial electrospinning [146]. The fibers were used for obtaining electrospun mats that exhibited wound healing properties in the case of diabetes-related ulcers and burns.

Another type of keratin-based hydrogel was obtained using tetraethyl orthosilicate and keratin extracted from bovine hooves. The keratin-silica hydrogel has a very porous microarchitecture and interesting properties, such as swelling capacity, optimal hardness, and spreadability [131]. This hydrogel showed good biocompatibility and can be used for wound healing.

In another research paper, the authors claimed the preparation of high molecular keratin (120 kDa) by using keratinase to extract the soluble keratin from wool, followed by the improvement of the keratin’s molecular weight due to the activity of transglutaminase [135]. Thereafter, nanofiber mats were prepared by co-electrospinning in presence of poly(3-hydroxybutyric acid-*co*-3-hydroxyvaleric acid) and treated with silver nanoparticles. The nanofibrous mats showed antibacterial and biocompatibility properties and demonstrated to be good candidates for tissue engineering.

In the attempt to improve the mechanical properties of the electrospun nanomats, Guidotti et al. [154] mixed keratin with poly(butylene succinate) and observed that the mats also showed better thermal stability, swelling ability, and biodegradability. Moreover, the potential to function as scaffolds for cell growth and drug delivery systems [154].

High-quality keratin films were obtained by extraction from duck feathers by using urea and L-cysteine. The L-cysteine reinforced keratin films obtained through drying at room temperature showed good characteristics for biomedical applications [132].

In the attempt to improve the characteristics of applied wound bandages, Zhai et al. [143] prepared a lyophilized hydrogel based on keratin, chitosan, and nano-ZnO [143]. The hydrogel assured a chilling effect along with a humid atmosphere, while the presence of the ZnO nanoparticles promoted the bactericidal effect. This porous nanocomposite showed good biocompatibility, while the in vivo studies on rats showed that wound healing is attained in a shorter time in comparison to the bare wound.

Another interesting application of keratin is to use it for materials that allow drug delivery. For example, Posati et al. have prepared keratin-based films by using nanosized ZnAl hydrotalcite-type material in which intercalated diclofenac in anionic form. The obtained hybrid films supported a controlled drug delivery for wound healing and indicated the possibility to use these films for tissue engineering applications [134].

Another possibility to valorize keratin was presented by Deng et al. [144], who blended it with polyethylene glycol, polylactic acid, and chitosan and obtained sutures through the hot-melt extrusion. These polymeric sutures were loaded with diclofenac potassium and characterized for their physical, mechanical, and thermal properties as well as for their drug-eluting capacity. Even though, the diclofenac-loaded sutures need further in vivo studies to prove their potential use in humans.

### 4.3. Environmental Applications

In general, the wastes rich in keratin are not safely disposed of, and most often this type of waste is either burned or dumped in the environment which can cause a serious impact on soil and groundwater [22]. A good solution for the valorization of keratin-rich wastes is to obtain materials that have adsorbing capacity and that can be used for the removal of other recalcitrant pollutants from the environment. In this respect, Hussain et al. have extracted keratin from wool and after lyophilization was cross-linked with glutaraldehyde. The form 3D keratin-glutaraldehyde sponge was successfully used for the removal of chromium ions that were present in the wastewater from the leather industry [13].

Zahara et al. [149] have used poultry feathers that after the removal of the fats and waxes, were dried and, thereafter, were chemically modified to increase the number of functional groups on the adsorbent surface. The obtained materials were tested for their ability to remove heavy metals from the wastewater resulting from the energy-generating processes. The results showed that their prepared biopolymers are efficient for the removal of cadmium, arsenic, copper, cobalt, vanadium (v), zinc, and chromium.

Jin et al. [150] prepared a nanofiber membrane that contains keratin extracted from wool by using a reducing agent (tris(hydroxypropyl) phosphine. The extracted keratin was mixed with PET in hexafluoroisopropanol, and the resulted solution was electrospun for the obtaining of the composite nanofiber membranes. These membranes showed good adsorption capacity for chromium (IV), as the maximum adsorption capacity was three times higher in comparison with the membranes obtained with pure PET [150].

Song et al. [155] have used silk fibroin and keratin to produce composite films that could be used for the removal of reactive brilliant blue dye [155]. The dye removal efficiency is attributed to the mechanical property and a high number of adsorption sites. Another important advantage of this prepared adsorbent is that has a good recycling performance, as the adsorption efficiency remains high even after five cycles of use.

### 4.4. Agricultural Applications

Closing the loop of keratin-rich fibrous material in a biomimetic manner involves application in agriculture, animal husbandry, and plant cultivation. In animal husbandry, extracted/hydrolyzed keratin is an efficient strategy for recycling nutrients, including essential amino acids [30]. A combined treatment of feathers, thermo-chemical with sodium sulfate (75 g/L feathers, 2.1–3.6 g/L Na_2_SO_3_, 85 °C, 60 min) and autoclaving (133 °C, 2.4 bar, 60–90 min) was proven to increased feed quality of the feather [156]. A protein hydrolysate with high antioxidative potential and better digestibility was produced by solid-state fermentation with a new keratinolytic bacterial strains, *Bacillus pumilus* A1 (in a minimal medium with 50 g/L wool at 45 °C, pH 10.0, incubated for 48 h) [157]. A free amino acids mix made by extensive hydrolysis of poultry keratin proven to improves survival of whiteleg shrimp post larvae (*Litopenaeus vannamei*), experimentally infected with *Vibrio parahaemolyticus*, bearing and endotoxin producing acute hepatopancreatic necrosis disease or with white spot syndrome virus [158].

**Table 9:** Application of the extracted keratin for the production and/or formulation of the agricultural inputs.

Keratin Rich Side Stream	Manufacturing Process	Agricultural Application	Reference
Wool	Superheated water hydrolysis (170 °C, 60 min, solid to liquid ratio close to 1)	Nitrogen fertilizer	[159]
Wool	Alkaline hydrolysis (80 °C, 12% NaOH, 4 h, solid to liquid ratio 1:3) of the degreased wool	Nitrogen fertilizer	[15]
Feathers	Steam hydrolysis (135 °C, 90 min, solid to liquid ratio 2:1), followed by enzymatic hydrolysis (50 °C deg. 48–120 h, solid to liquid ratio 1:1)	Nitrogen fertilizer	[160]
Feathers	Coating produced from chicken feathers’ protein, acrylic acid, and *N*,*N*′-methylenebisacrylamide	Coating materials for controlled-release fertilizers; water retention/superadsorbent coating	[161]
Bovine hair	Keratin based superadsorbent coating produced from extracted keratin from waste bovine hair, acrylic acid, *N*,*N*-methylenebis acrylamide	Coating materials for controlled-release fertilizers; water retention/superadsorbent coating	[137]
Feathers	Solubilization of keratin in deep eutectic solvents (60 °C, 2 h, solid to liquid ratio 1:1), followed by dilution to water till 50% water and enzymatic hydrolysis with commercial protease (Alcalase, pH 8.2, 60 °C, 2 h). Resulted hydrolysate was coated into NPK fertilizer granules in a fluid bed granulator	Coating materials for controlled-release fertilizers	[162]
Bovine hair	Keratin extracted from bovine hair, included into a film-forming formulation, polyvinyl alcohol, polyacrylamide, *N*,*N*-methylenebis (acrylamide) and glycerol (GL) to prepare the low-cost degradable keratin-based sprayable mulch film	Sprayable mulch, with water holding capacity (380%) and high mineral nutrient content	[138]
Feathers	Supernatant produced by degradation of feathers by *Chryseobacterium* sp. RBT (accession number GU481093), grown in basalt salt medium and feathers, for 30 h, on rotary shaker (140 rpm) at 37 °C.	Plant biostimulant effects; quality improvement of banana produced by banana plant treated with feather hydrolysate	[163]
Feathers	Supernatant produced by degradation of feathers by *Trichoderma asperellum* T50 and *T. atroviride* grown in minimal medium and feathers, for 21 days, at 26 ± 2 °C and constant agitation—130 rpm	Plant biostimulant effects; activation of the proton pump, tomato seedling stimulation	[29]
Feathers	Supernatant produced by degradation of feathers by *Bacillus aerius* NSMk2 grown in a minimal media with 1.375%, pH 7.5, at 35 °C, for 3 days	Plant biostimulant effects; enhance mung bane growth and development	[164]

Applications of extracted keratin in agriculture are related mainly to the production and/or formulation of the agricultural inputs—Table 9.

The extracted/hydrolyzed keratin from keratin-rich waste was mainly used for the production of organic fertilizers and/or plant biostimulants and as a coating material for controlled released fertilizers. The wool was hydrolyzed till amino acids by superheated water and the resulted hydrolysate was proposed to be used as nitrogen fertilizers, including for grazing pastures [165]. Alkaline hydrolysis was also proposed for the production of nitrogen fertilizer based on amino acids [15].

Plant biostimulants based on hydrolyzed keratin exploit the bioactivity of the amino acids released from keratin, also in combination with bioactive compounds released by the keratinolytic microorganisms, bacteria, or fungi. Plant biostimulants are a new category of agricultural inputs, which enhance/benefit nutrient uptake, increase plant tolerance to abiotic stress, and improve edible yield quality. Application of AminoPrim and AminoHort, two plant biostimulants containing 15% and 20% amino acids derived from chicken feathers, respectively, at a dose of 1.0 Lha^−1^ and 1.2 Lha^−1^, respectively, increase the grain yield of winter wheat by 5.4% and by 11%, respectively [166]. Quality indicators of the wheat grains produced by treated plants, such as protein content and Zeleny sedimentation index, were also improved. Keratin hydrolysate produced by *Bacillus aerius* NSMk2 (bacterial metabolites) enhanced mung beans (*Vigna radiata*) growth (better germination and better plant growth) and development (more flowers, more pods, and more seeds) [164]. Keratin hydrolysate produced by two *Trichoderma* strains showed an activation of the proton pump (and, therefore, stimulation of nutrient uptake by roots) and promoted the development of the tomato (*Solanum lycopersicum*) seedlings [29].

Extracted/hydrolyzed keratin was also used as a coating for controlled released fertilizer. An interesting result was obtained by Chen et al. [137] that developed a fertilizer based on extracted keratin from bovine hair. The extracted keratin was combined with acrylic acid and *N*,*N*-methylene bis acrylamide. The obtained keratin-superabsorbent was used as a coating material for the ethylcellulose that incorporates urea particles. The obtained fertilizer proved to act as a slow-release reservoir for urea and improved the water retention of soil. An important property of this fertilizer was also observed in regards to the inhibition of the chromium (III) in the soil. This fertilizer incorporating keratin can be considered that contributes to the safety of the food industry and sustainable development in agriculture.

Keratin extracted from bovine hair, by using spent liquor from a pulp factory, was used for the formation of a sprayable mulch composition, with high water holding capacity (380%) and high mineral nutrient content (from pulping spent liquor). Biodegradable sprayable mulch is a sustainable alternative to oil-derived plastic mulch [167], improves water management in the soil during vegetable cultivation [168], and represents an eco-efficient mean to control weeds [169].

### 4.5. Emerging Applications

Besides the above-mentioned applications of keratin, in the literature are also reported interesting applications that could be successfully used.

The keratin hydrolysate was used to assist and stabilize preparations of metallic nanoparticles. Agarwal et al. [151] recently reported that keratin extracted from human hair was used as an exfoliation liquid and obtained a highly concentrated colloid of stable single-layered MoS2 nanosheets. In comparison to the classical liquid exfoliation methods, that need further processing steps, their proposed method is facile, efficient, and leads to higher yields. Moreover, the obtained nanosheets have a long shelf-life and good electronic properties [151]. Silver nanoparticles with an average diameter 3.5 nm were stabilized by using wool keratin hydrolysates. The stabilized nanoparticles retained their anti-microbial activity after freeze-drying [170]. Selenium nanoparticles with controlled size produced by keratin-mediated synthesis at 65 °C protect zebrafish embryos and cardiomyoblasts against oxidative stress induced by ethanol [171].

The possibility of producing co(polymers) that can be used for 3D and 4D printing responsive materials without any chemical’s addition was explored by Grigsby and his group [12]. For this purpose, they combined keratin and lignin that were cross-linked in an aqueous solution at an acidic pH. The keratin-lignin copolymer materials can be used for the preparation of 3D paste printing and showed to have a good potential as smart materials for 4D applications.

## 5. Perspectives on Utilization of Extracted Keratin-Rich Fibrous Materials

Eco-efficient and profitable loop closing of the keratin-rich fibrous materials also involves the utilization of the extracted/spent keratin-rich fibrous materials. γ-keratin is less soluble during enzymatic extraction of keratin-rich fibrous material [42]. Further utilization for biomedical and/or cosmetic purposes of such enzymatic extracted keratin-reach material is due to the healing characteristics brought by the presence of γ-keratin [45,142].

**Figure 4:**
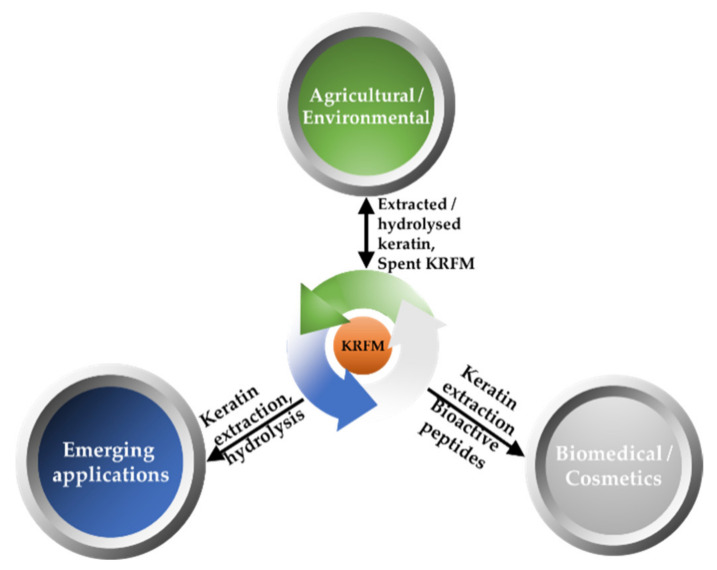
Circular utilization of the keratin-rich fibrous material (KRFM). The emerging application of extracted/hydrolyzed keratin is related to 3D and 4D printing, and stabilization of metallic nanoparticles. Cosmetic and biomedical applications are related to the high biocompatibility of keratin biomaterials, bioactive peptides derived from keratin, healing promoting ability of γ-keratin. Agricultural and environmental applications benefit from film-forming and water holding abilities of KRFM.

Extraction of the wool by oxidation determines the formation of thick jelled material composed mainly of β-keratin [23]. Such a utilization should close the loop in a circular manner, returning nutrients to primary production systems in a “smart” manner, exploiting film forming and water holding abilities of spent keratin-rich fibrous material (KRMF)—Figure 4.

Examples of such “smart environmental applications of recalcitrant/spent KRMF are production of materials intended to prevent soil erosion and/or to support soil fertility. Geotextile meandrically arranged rope was produced from strips of needle-punched nonwoven wool—Kemafil technology [172,173]. Such rope, which proved to be very efficient in controlling soil erosion [174], could be produced also from KRMF extracted for α-keratin. The remaining insoluble β-keratin should be a longer-lasting material.

Wool hydrolyzed by super-heated water was proposed to be used together with biochar produced from lignocellulose-rich agro-industrial side streams to enhance soil fertility [175]. Hydrolyzation by super-heated water should be effective also for KRMF residues, by-products of softer extraction methods (such as enzymatic or oxidative methods). Lignocellulose-rich residues were used also to produce pellets for soil treatment, with plant biostimulant effect [176]. This pelletizing technology is useful also for KRMF residues. An additional benefit of using keratin-rich material for soil treatment is related to the development of microbial communities which antagonize the soil-born plant pathogens [177].

## 6. Conclusions

This review has in attention the possibilities of closing the loop with keratin-rich fibrous materials. KRFM becomes costly if is treated as a waste, but valorization of these by-products through the eco-friendly extraction methods of the keratin might turn it into a valuable resource for the obtaining of new materials of great importance in different industries. The different extraction methods described in this review open the possibility to use keratin for various purposes.

The chemical, enzymatic, and physical methods for keratin extraction are discussed along with their main disadvantages. Moreover, the possibilities of using the extracted keratin for cosmetics, biomedical, agricultural, and environmental applications are also detailed. It has to be mentioned that depending on the extraction method, low molecular keratin can be successfully used for cosmetic products, while through other extraction methods, the keratin can be combined with other materials to obtain new products that can be used for biomedical applications.

Moreover, the interesting use of keratin is for the fabrication of adsorbents that can be successfully used for the removal of some pollutants from the environment. Agricultural applications of extracted/hydrolyzed keratin include its use as ingredients for feeds, plant biofertilizers/plant biostimulants, soil amendments, and formulation additives for agricultural inputs.

Nevertheless, recent studies proved that keratin was used for obtaining new materials with improved properties, but further studies are necessary, especially for the development of efficient extraction of the desired type of keratin with a yield that can satisfy its use in an industrial process. Special attention should be paid to the compatibility of the used extraction method with the desired application, as in some cases this might have a negative impact on the further use of the extracted keratin.

## Figures and Tables

**Table 1:** Structure of α-keratin and β-keratin adapted from [7].

Property	α-Keratin	β-Keratin
Filaments’ diameters	IFs: ~7 nm	β-keratin filaments: 3–4 nm
Constitutive proteins	IFs: several low-sulfur proteins Matrix: high-sulfur and high-glycine-tyrosine proteins	Presents no different types of proteins The filament and matrix are integrated into one single protein
Specific structure	Based on α-helical conformation	Based on β-plated sheet arrangement
Molecular weight	40–68 kDa	10–22 kDa

**Table 2:** Oxidation conditions and processing parameters for the keratin extraction.

Keratin Source	Processing Parameters		Reference
	Oxidative Agent	Conditions	
Wool	Peracetic acid 2%	37 °C; overnight, 180 rpm	[74]
Wool	Solid sodium percarbonate 4.5% mass ratio Fiber, liquid ratio 1:35 NaOH 30%	Room temperature; 3–4 h stirring	[74]
Wool	Peracetic acid 24% Fiber, liquid ratio 1:60	2 days Room temperature Yield 57%	[23]
Wool	Peracetic acid 2% Fiber, liquid ratio 1:50	25 °C; 24 h	[75]
Human hair	Performic acid (100 volume H_2_0_2_/98% formic acid (1:39 *v*/*v*)	4 °C; 18 h	[70]
Human hair	Peracetic acid 2% Hair mass, liquid ratio 1:20	37 °C; 10 h, 150 rpm	[32]
Human hair	Peracetic acid 2.5%	Room temperature; Overnight	[76]
Human hair	Thioglycolic acid 0.5 M;	37 °C; 15 h; pH 10.4	[76]

**Table 3:** Optimized reductive extraction conditions and processing parameters used for the keratin extraction.

Keratin Source	Processing Parameters		Reference
	Reductive Agent	Conditions	
Wool	Extractive liquor: thiourea 2.6 M; urea 5 M; 2-mercaptoethanol 5%	50 °C; 3 days; pH 8.5, 100 rpm	[74]
Wool	Extractive liquor: urea 8 M; SDS 0.25%; 2 g sodium metabisulfite Fiber to liquid ratio > 1:70	65 °C; 5 h, 120 rpm	[74]
Wool	Extractive liquor: urea 8 M; Tris * 0.5 M; DTT **, 0.14 M; ethylenediaminetetraacetic acid EDTA, 6 mM. Fiber to liquid ratio 1:25	25 °C; 2.5 h; pH 8.6	[81]
Feathers	Extractive liquor: sodium hydroxide 1.78%; sodium bisulfite 0.5% Fiber to liquid ratio 1:5	87 °C; 111 min Yield 68.2	[17]
Wool (red sheep hair)	Extractive liquor: urea 8 M; SDS 0.26 M; mercaptoethanol 1.66 M Fiber to liquid ratio 1:20	50 °C; 12 h, under shaking	[82]
Wool	Extractive liquor: urea 7 M, thiourea 2 M, Tris 50 mM, TCEP 50 mM Fiber to liquid ratio 10:1	18 h; pH 4.3	[80]

* tris(hydroxymethyl)aminomethane; ** dithiothreitol.

**Table 4:** Sulfitolysis conditions and processing parameters used for the keratin extraction.

Keratin Source	Processing Parameters		Reference
	Extracting Solution	Conditions	
Chicken Feather	Extractive liquor: sodium metabisulfite 0.2 M; urea 8 M; SDS 0.6 g/g feather Fiber to liquid ratio 1:35	65 °C; 5 h; pH 6.5 Yield 87.6%	[84]
Hair	Sodium sulfide 0.125 M	40 °C; 4.5 h	[76]
Wool	Extractive liquor: urea 8 M; sodium metabisulfite 0.5 M Fiber to liquid ratio 1:25	65 °C; 2.5 h; pH 7;	[81]
Wool (red sheep hair)	Extractive liquor: urea 8 M; sodium metabisulfite 0.5 M Fiber to liquid ratio 1:20	65 °C; 2 h constant stirring	[82]

**Table 5:** Alkaline conditions and processing parameters used for the keratin extraction.

Keratin Source	Processing Parameters		Reference
	Extracting Solution	Conditions	
Wool	KOH; CaO 5% 10% 15%	140 °C/170 °C/1 h	[88]
Wool (red sheep hair)	Extractive liquor: NaOH 0.5 N Fiber to liquid ratio 1:20	60 °C; 3 h	[82]
Feathers	NaOH 0.75 N;	60 °C; 45 min Yield 82%	[62]

**Table 6:** Ionic liquids and processing parameters used for the keratin extraction.

Keratin Source	Processing Parameters		Reference
	Extracting Solution	Conditions	
Feathers	[Choline][thioglycolate] Solid, liquid ratio: 1:2	130 °C; 10 h Solubility up to 45%	[94]
Feathers	[Bmim]Cl Solid, liquid ratio: 1:2	130 °C; 10 h Solubility up to 51%	[94]
Feathers	[Amim]Cl Solid, liquid ratio: 1:2	130 °C; 10 h Solubility up to 51%	[94]
Feathers	[BMIN]Cl + 10% wt Na_2_SO_3_ Solid:liquid ratio: 1:20	90 °C; 60 min Keratin yields: 75.1%	[96]
Wool	[DBNE]DEP Solid, liquid ratio: 8 wt%	393 K, 3 h Yield: 0.447 g/g	[97]
Wool	[DBNM]DMP Solid, liquid ratio: 8 wt%	393 K, 3.5 h Yield: 0.4 g/g	[97]
Feathers	[Bmim]Cl 500 mg in 20 g	130 °C; 2 h	[98]
Feathers	[Bmim]Cl: dimethylsulfoxide mixture 35:65 500 mg in 20 g Ultrasonic treatment: 200 W	52 min	[98]
Feathers	[Bmim]Cl 500 mg in 20 g Ultrasonic treatment: 200 W	20 min	[98]

**Table 7:** Keratinolytic microorganisms and degradation conditions of keratin substrates.

Microorganism	Substrate	Degradation Condition		Reference
		pH	Temperature (°C)	
*Bacillaceae*				
*Bacillus subtilis*	Feathers, human hair	9	50	[109]
*Bacillus cereus Wu2*	Feathers	7	30	[110]
*Bacillus* sp. FPF-1	Feathers	5	25	[111]
*Bacillus pumilus*	Bovine hair	8	35	[112]
*Brevibacillus brevis US575*	Feathers, hair	8	55	[113]
Gram negative bacteria				
*Meiothermus sp. 140*	Feathers, wool, hair	8	70	[114]
*Stenotrophomonas maltophilia BBE11-1*	Feathers, wool	9 (7–11)	40–60	[115]
*Vibrio sp. kr2*	Feathers	6–8	30	[34]
*Actinobacteria*				
*Streptomyces gulbargensi*	Feathers	8 (7–9)	45 (30–60)	[116]
*Aphanoascus fulvescens* *Chrysosporium articulatum*	Feathers	7.5	28.7	[117]
*Fungi*				
*Aspergillus niger*	Feathers, human hair, sheep’s wool	5	-	[118]
*Purpureocillium lilacinum*	Hair	6 (4–9)	60 (20–65)	[119]
*Trichopyton sp. HA-2*	Chicken feathers	8	35	[120]
*Trichoderma asperellum*, *Trichoderma atroviridae*	Feathers	7.5	26	[29]
*Fusarium sp. 1A*	Horse hair	7.5	27	[121]

**Table 8:** Optimal processing parameters for keratin extraction by steam explosion method.

Keratin Source	Processing Parameters			Reference
	Temperature (°C)	Pressure (MPa)	Time (min)	
Wool	164.2	0.2–0.6	2–8	[124]
Feathers	Saturated steam	1.6	1	[125]
Feathers	Saturated steam	1.8–2	1	[126]
